# Supplementary material for: Accuracy of Speech-to-Text Transcription in a Digital Cognitive Assessment for Older Adults
Source: Brain Sci. 2025 Oct 9;15(10):1090. doi: 10.3390/brainsci15101090 (PMC12563218; doi:10.3390/brainsci15101090)
Supplement: Supplementary file 1 [file brainsci-15-01090-s001.zip › brainsci-3877560-supplementary.pdf]

Supplementary Table S1: Example STT Corrections

Original transcription	Postprocessed transcription
**Plurals**	
motorcycles	motorcycle
bookcases	bookcase
cabbages	cabbage
giraffes	giraffe
**Common mistranscriptions**	
motor cycle	motorcycle
bouquets	bookcase
spanish	spinach
swirl	squirrel
katz	cats
